# Differential Gene Expression in the Prefrontal Cortex and Hippocampus Following Long-Access Methamphetamine Self-Administration in Male Rats

**DOI:** 10.3390/ijms26041400

**Published:** 2025-02-07

**Authors:** Christopher L. Robison, Victoria Madore, Nicole Cova, Mona Karbalivand, Sherine F. Elsawa, Sergios Charntikov

**Affiliations:** 1 Department of Psychology, University of New Hampshire, Durham, NH 03824, USA; 2 Molecular, Cellular, and Biomedical Sciences, University of New Hampshire, Durham, NH 03824, USAsherine.elsawa@unh.edu (S.F.E.)

**Keywords:** methamphetamine self-administration, differential gene expression, prefrontal cortex, synaptic plasticity, behavioral economics

## Abstract

Methamphetamine (METH) is a potent psychostimulant that disrupts cognitive and neurobiological functions in brain regions such as the prefrontal cortex (PFC) and hippocampus. Chronic METH use leads to altered synaptic plasticity, neuroinflammation, and mitochondrial dysfunction, contributing to methamphetamine use disorder (MUD). This study investigates gene expression changes following long-access intravenous METH self-administration in a rodent model. RNA sequencing (RNA-Seq) was conducted on PFC and hippocampal tissue to identify differentially expressed genes (DEGs) between METH-treated and control groups. We identified 41 DEGs in the PFC and 32 in the hippocampus, many involved in synaptic plasticity, immune response, and energy metabolism. Key findings included downregulation of mitochondrial function genes and upregulation of genes related to neural development and extracellular matrix organization, highlighting the profound transcriptional effects of METH. As a proof-of-concept, we explored individual gene expression variability in relation to economic demand for METH. Rats exhibiting higher demand showed distinct molecular profiles, including upregulation of genes linked to neural signaling and transcription regulation, such as *Foxd1* and *Cdh1*. This preliminary analysis demonstrates that individual differences in drug-seeking correlate with unique gene expression patterns. These findings suggest that both group-level and individual molecular changes contribute to the neurobiological mechanisms of METH use. A better understanding of these individual differences could potentially inform the development of personalized therapeutic approaches for MUD.

## 1. Introduction

Methamphetamine (METH) is a powerful psychostimulant with severe neurotoxic effects that lead to significant alterations in brain structure and function [1]. METH is associated with long-term neurobiological changes, cognitive deficits, and high rates of use [2]. METH use disorder (MUD) remains a pressing public health concern due to its widespread use and limited treatment options. In the brain, METH affects key areas such as the prefrontal cortex (PFC), hippocampus, and striatum (among others) —regions critical for decision-making, learning, and memory [3]. Repeated METH exposure has been shown to induce widespread transcriptional changes in these regions, affecting pathways involved in synaptic plasticity, immune response, oxidative stress, and mitochondrial dysfunction [4,5,6,7]. These transcriptomic alterations are believed to underlie the behavioral and cognitive deficits observed in chronic METH users. While much is known about the neurobiological effects of METH at the group level, there is a growing recognition of the need for more individualized approaches that consider the variability in genetic, behavioral, and neurobiological responses to drug use. In this context, transcriptomic approaches such as RNA sequencing (RNA-Seq) have become valuable tools for unraveling the complex molecular mechanisms of substance use by enabling high-throughput, unbiased analysis of gene expression changes induced by METH in key brain regions.

Previous research has identified a wide range of gene expression changes associated with METH use, particularly in brain regions such as the prefrontal cortex and hippocampus, which are heavily implicated in substance use-related behaviors. Transcriptomic approaches have revealed that METH alters the expression of genes involved in crucial biological processes like synaptic plasticity, immune responses, and neuroinflammation [3]. For example, in the PFC, single-nucleus RNA sequencing of tissue from METH-treated mice revealed altered gene expression primarily in genes related to mitochondrial function, adenosine triphosphate (ATP) metabolism, oxidative phosphorylation, and myelin sheath production [8]. Another study investigating gene expression in the PFC of mice that were voluntarily drinking METH solution found differential gene expression related to glutamatergic and GABAergic neurotransmission, as well as synaptic plasticity [9]. In the hippocampus of rats treated with non-contingent METH, RNA-seq analysis revealed altered expression of 1210 genes in pathways related to cell proliferation, senescence, synaptic transmission, and signaling mechanisms, including oxytocin, axon guidance, cAMP, Wnt, endocannabinoid, and long-term potentiation [10]. Furthermore, a study using a rodent model of compulsive methamphetamine self-administration in the presence of adverse consequences (foot shocks) found differential expression genes in the hippocampus related to cell adhesion [11]. These studies collectively underscore the profound and multifaceted impact of methamphetamine on gene expression across critical brain regions. The alterations in genes related to synaptic plasticity, neurotransmission, and cellular metabolism suggest that METH use may lead to long-lasting changes in neural circuitry and function. Despite these advancements, most research in this area has focused on group-level effects, potentially masking individual variations in molecular responses to METH. Understanding how individual differences in drug-taking behavior correlate with distinct gene expression profiles is an emerging focus, as these differences could inform personalized interventions for substance use. This shift towards incorporating individual variability in gene expression studies is particularly relevant given the heterogeneous nature of substance use, where individuals exhibit varying degrees of vulnerability and resilience to METH-induced changes at both molecular and behavioral levels.

Despite extensive research into METH-induced gene expression changes, significant gaps remain in understanding how the progression of MUD aligns with underlying molecular alterations. While previous studies have provided valuable insights into METH’s effects on gene expression in various regions of the rodent brain, many of these investigations relied on non-contingent methods of METH administration [5,12,13,14,15,16,17,18,19] or short-access self-administration protocols, e.g., [20]. Given this, it is essential to model relevant drug administration patterns when investigating the neurobiological substrates of methamphetamine (METH) use to ensure relevant findings. Intravenous self-administration, in particular, closely mirrors human consumption patterns, providing a more relevant model for studying methamphetamine use. Moreover, while short-access protocols (1–2 h) are favored for their efficiency and high throughput, long-access protocols (6 h or more) more accurately replicate the daily drug intake patterns observed in humans [21,22,23]. In addition to modeling drug intake patterns, self-administration paradigms enable the assessment of motivation for a substance with the help of reinforcer demand modeling. The reinforcer demand approach, adapted from microeconomic theory, examines how the cost of obtaining a reinforcer (e.g., food, drugs) impacts consumption [24,25]. In behavioral studies, rats are trained to work for a reinforcer on a progressively increasing fixed ratio (FR) schedule, where the ‘cost’ increases across daily sessions. In this framework, the reinforcer is considered a ‘good’, the effort to obtain it represents ‘consumption expenditure’, and the FR schedule reflects ‘cost’. This methodology, which has been refined in prior work from our laboratory, can be used to offer insights into grouped and individual substance use patterns, including nicotine, ethanol, and heroin [26,27,28,29,30]. This refined methodology, combining long-access intravenous self-administration protocols with reinforcer demand modeling, enables a more accurate assessment of both group and individual substance use behaviors, providing critical insights into the molecular mechanisms underlying MUD.

Thus, the primary objective of this study was to investigate how METH self-administration impacts gene expression in the prefrontal cortex (PFC) and hippocampus, with a focus on both group-level effects and individual variability. Specifically, we utilize a long-access model of METH self-administration to examine gene expression changes in these brain regions, providing a more accurate reflection of human substance-use patterns. As a proof-of-concept, we also explored whether individual differences in economic demand for METH—a measure of motivation and drug-seeking behavior—can serve as predictors of gene expression variability. Here, high economic demand is conceptualized as a phenotype indicating increased vulnerability to METH use. By integrating behavioral economics with transcriptomic data, we hypothesize that METH exposure will result in significant differential expression of genes related to synaptic plasticity, neuroinflammation, and metabolic processes in both the PFC and hippocampus. Moreover, we expect that individual differences in economic demand will correlate with distinct gene expression profiles, highlighting molecular signatures associated with vulnerability to METH use. This dual approach may not only reveal group-level molecular changes but may also identify individual-specific alterations, offering deeper insights into the neurobiological mechanisms underlying methamphetamine use disorder.

## 2. Results

### 2.1. Acquisition and Economic Demand Analysis of METH Self-Administration

#### 2.1.1. Acquisition of METH Self-Administration

Over the first seven sessions of self-administration, rats that had access to METH had significantly more infusions than rats that had access to saline on sessions 2–7 [main effect of Drug; F(1, 80) = 202.71, *p* < 0.001; Bonferroni comparisons)]. It was found that the main effect was from Session [F(1, 80) = 38.17, *p* < 0.001)] and Group by Session interaction [F(1, 80) = 34.77, *p* < 0.001)]. Rats that had access to METH escalated their intake from 45.66 infusions in session 1 (2.28 mg/kg; SD = 52.78) to 258.83 infusions in session 7 (12.94 mg/kg; SD = 55.60). Rats that had access to saline slightly increased their intake from 13.50 infusions in session 1 (SD = 3.14) to 18.33 infusions in session 7 (SD = 5.53). For the METH condition, a separate ANOVA revealed main effects of Lever [F(1, 80) = 21.84, *p* < 0.001], Session [(F(1, 80) = 13.74, *p* < 0.001], and their interaction [F(1, 80) = 5.94, *p* < 0.05]. The number of active lever presses in the METH condition was significantly higher than inactive in sessions 2–7 (Bonferroni comparisons). For the saline condition, a separate ANOVA revealed the main effect of Lever [F(1, 80) = 188.56, *p* < 0.001], no effect of Session (*p* = 0.56), and a significant interaction [F(1, 80) = 4.33, *p* < 0.05]. The number of active lever presses in the saline condition was significantly higher than inactive on sessions 1–7 (Bonferroni comparisons).

#### 2.1.2. Economic Demand Comparisons

**Figure 1:**
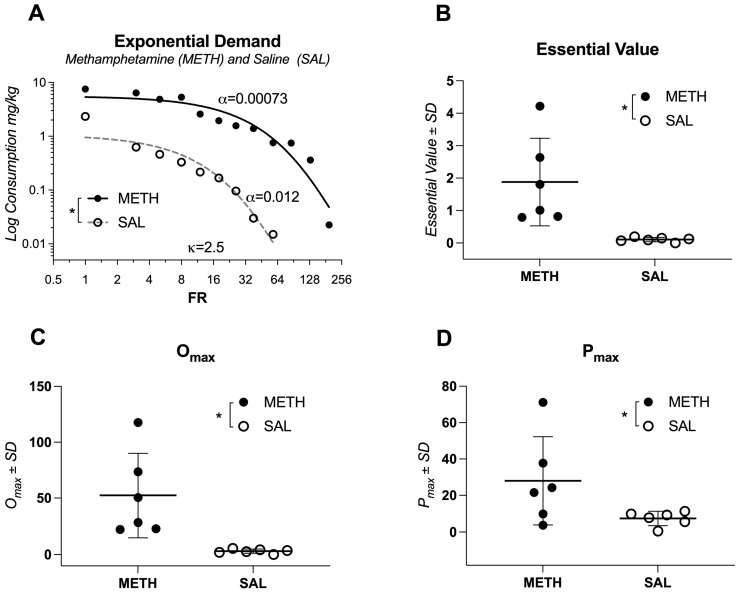
Behavioral analysis of methamphetamine self-administration in rats. (**A**) Comparison of individual demand curves for methamphetamine (METH) and saline conditions, highlighting significant differences in motivation levels (*p* < 0.0001). (**B**) Economic demand analysis shows that METH-treated rats worked significantly harder for METH infusions (*Essential Value*, *EV*). (**C**) Maximum expenditure (O_max_) for METH was higher compared to saline, indicating greater reinforcement value. (**D**) No significant difference in *P_max_*, the maximum effort exerted for reinforcement, was observed between groups. Data represent mean values, and error bars indicate standard deviation. * Denotes significant differences between the groups.

Table 1 presents sample information and behavioral economics parameters for each subject, which can be referenced alongside Figures and Supplementary Data Files for transparency and to enable independent analyses. Table 2 summarizes the results of *t*-tests comparing behavioral economics parameters between the two groups. A least squares regression analysis comparing individual fit for each condition showed significant differences between rats self-administering METH or saline (alpha different for each dataset; F(1, 16) = 75, *p* < 0.0001; Figure 1A; compare two curves). Analyses of additional parameters from the reinforcer demand model revealed that rats with access to METH earned more reinforcers at a simulated zero price (*Q*_0_), worked harder overall for METH infusions (*EV*; Figure 1B), and exhibited higher maximal expenditure sustained by a reinforcer (*O_max_*; Figure 1C). However, there was no significant difference in *P_max_*, the maximum effort exerted for a reinforcer (see output of all relevant *t*-tests in Table 2; Figure 1D). Finally, rats that had access to METH showed a high degree of variability in their sensitivity to changes in cost (*EV*; Figure 1B), which is important for studies focusing on individual effects as it increases statistical power to detect individual effects using regression types of analyses.

#### 2.1.3. Reacquisition of Self-Administration

Over the last seven sessions of self-administration, rats that had access to METH had significantly more infusions than rats that had access to saline across all sessions [main effect of Drug; F(1, 80) = 423.45, *p* < 0.001; Bonferroni comparisons)]. There was no main effect of Session (*p* = 0.70) and no Group by Session interaction (*p* = 0.85). Rats that had access to METH received on average 172.19 infusions (8.61 mg/kg) while rats that had access to saline received on average 16.26 infusions. For the METH condition, a separate ANOVA revealed the main effects of Lever [F(1, 80) = 290.28, *p* < 0.001], no effect of Session (*p* = 0.15) and no interaction (*p* = 0.15). The number of active lever presses in the METH condition was significantly higher than inactive across all sessions (Bonferroni comparisons). For the saline condition, a separate ANOVA revealed a main effect of Lever [F(1, 80) = 221.94, *p* < 0.001], no effect of session (*p* = 0.77), and no interaction (*p* = 0.21). The number of active lever presses in saline condition was significantly higher than inactive across all sessions (Bonferroni comparisons).

### 2.2. Transcriptomic Changes in the PFC: Differential Gene Expression

Our RNA-Seq analysis identified 41 significantly differentially expressed genes (DEGs) in the prefrontal cortex between methamphetamine-treated and control groups. Of these, most were protein-coding genes, with a small percentage comprising non-coding RNA species such as lincRNAs and miRNAs. Notable downregulated genes included *AABR07066510* (log_2_(fc) = −5.98) and *LOC100910732* (log_2_(fc) = −4.84), while *AABR07015081* (log_2_(fc) = 1.80) and *AABR07015066* (log_2_(fc) = 1.66) were among the significantly upregulated genes (see Supplemental File PFC_SALVSPFC_METH_Gene_differential_expression.csv for a complete list of DEGs with fold changes and *p*-values).

**Figure 2:**
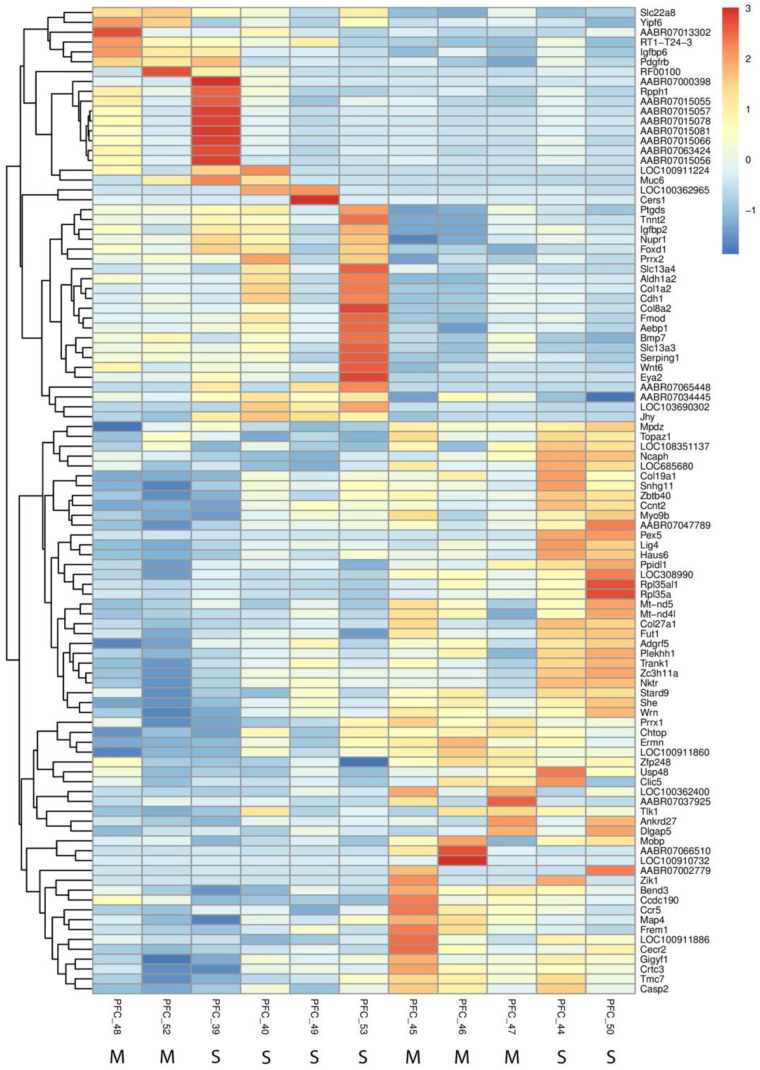
Heatmap of differentially expressed genes in the prefrontal cortex following methamphetamine self-administration. The heatmap displays the most significantly altered genes between methamphetamine-treated and control groups, highlighting upregulated and downregulated genes. Each column represents an individual sample, and each row represents a specific gene. Color intensity indicates the magnitude of expression change, with upregulation in red and downregulation in blue. At the bottom of the figure, “M” indicates a sample from the METH group, and “S” indicates a sample from the saline group.

Functional annotations revealed that methamphetamine altered several biological processes. Transcriptional regulators such as *Foxd1* (log_2_(fc) = 1.08) and *Prrx2* (log_2_(fc) = 1.15), both of which are implicated in neural development and differentiation, were notably upregulated. Additionally, genes associated with extracellular matrix organization, such as *Col1a2* (log_2_(fc) = 1.01), and immune response genes like *RT1-T24-3* (log_2_(fc) = 1.12), were significantly altered. Furthermore, methamphetamine exposure significantly downregulated genes related to mitochondrial and peroxisomal function, particularly *Pex5* (log_2_(fc) = −14.82), a key component in peroxisomal protein import. This drastic downregulation suggests that methamphetamine disrupts metabolic processes, possibly contributing to oxidative stress. Additionally, genes involved in ribosomal function and protein synthesis were affected, with *Rpl35a* (log_2_(fc) = −1.28) and *Rpl35al1* (log_2_(fc) = −1.64) showing marked downregulation. These alterations could reflect impairments in neuronal protein synthesis machinery, potentially affecting cell viability. These transcriptomic alterations are visually summarized in the heatmap (Figure 2), which highlights the most significantly changed genes.

### 2.3. Differential Gene Expression in the Prefrontal Cortex: Insights from Gene Ontology Enrichment Following Methamphetamine Exposure

**Figure 3:**
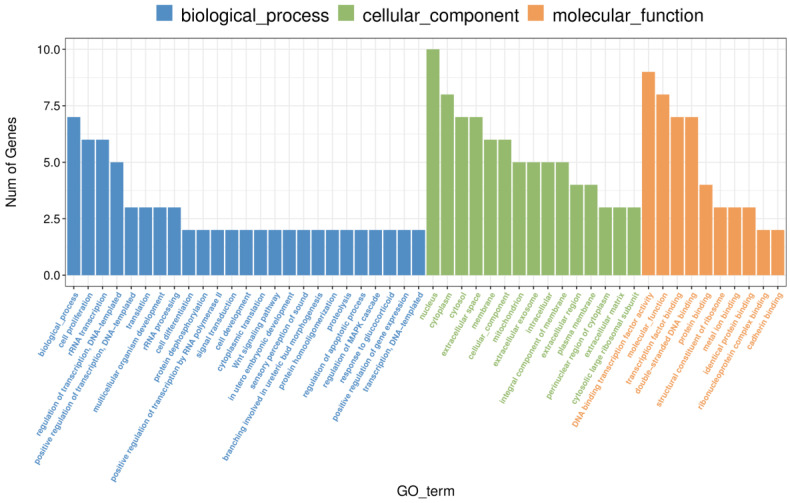
Gene ontology (GO) enrichment analysis of differentially expressed genes in the prefrontal cortex following methamphetamine exposure. The bar chart illustrates significantly enriched GO terms across biological processes, cellular components, and molecular functions for differentially expressed genes. Each bar represents a GO term, with the length corresponding to the number of genes.

To further investigate the biological implications of methamphetamine-induced gene expression changes, we performed Gene Ontology (GO) enrichment analysis on differentially expressed genes (DEGs) identified in the prefrontal cortex. Significant enrichment was observed in several GO terms across three categories: biological processes, molecular functions, and cellular components (see Supplemental File PFC_SALVSPFC_METH_GO_enrichment_Gene.csv for a complete list of results). In the biological process category, “rRNA transcription” (GO:0009303) was prominently enriched, driven by the upregulation of six genes (*AABR07015066*, *AABR07015081*, *AABR07015078*, *AABR07015055*, *AABR07015057*, and *AABR07063424*). These changes suggest that methamphetamine enhances ribosomal RNA synthesis, potentially boosting protein synthesis capacity. Additionally, methamphetamine exposure significantly impacted DNA-related processes, with “double-stranded DNA binding” (GO:0003690) and “transcription factor binding” (GO:0008134) enriched in upregulated genes such as *Foxd1*, *LOC103690302*, and *AABR07015081*. The consistent upregulation of transcription factor genes implies a coordinated effect on transcriptional regulation. Notably, the term “cell proliferation” (GO:0008283) was enriched, with key genes showing upregulation, including *AABR07015066* and *AABR07063424*, indicating methamphetamine’s influence on promoting cell proliferation. Overall, these GO enrichments reveal that methamphetamine alters fundamental processes in the prefrontal cortex, including transcription regulation, DNA binding, and cell proliferation. Figure 3 provides a comprehensive visual summary of the enriched biological processes, further supporting the molecular alterations caused by methamphetamine exposure.

### 2.4. Comparative Analysis of KEGG Pathway Enrichment in the Prefrontal Cortex: Methamphetamine Versus Saline Treatment

**Figure 4:**
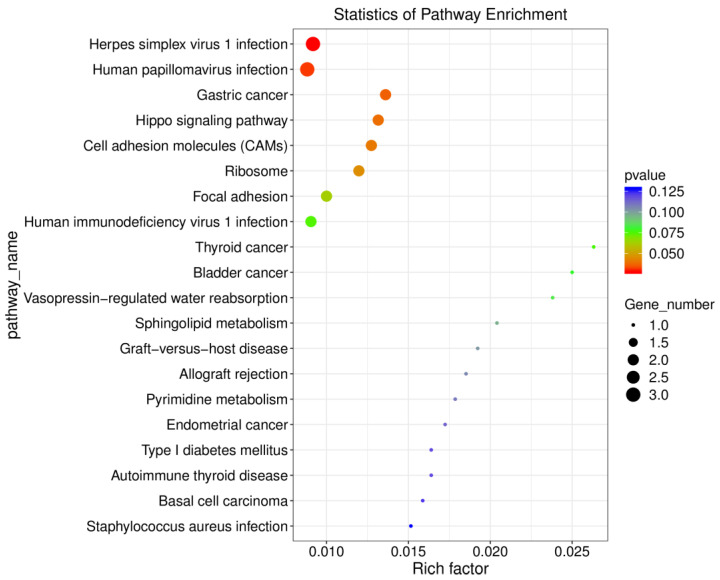
KEGG pathway enrichment analysis of differentially expressed genes in the prefrontal cortex following methamphetamine exposure. The chart depicts significantly enriched KEGG pathways in methamphetamine-treated rats compared to controls. The number of genes in each pathway is represented by the point size, and the color reflects the *p*-value.

To further explore the functional implications of methamphetamine-induced gene expression changes, we performed KEGG pathway enrichment analysis on differentially expressed genes (DEGs) in the prefrontal cortex (see Supplemental File PFC_SALVSPFC_METH_KEGG_enrichment_Gene.csv for a complete output from these analyses). Several pathways were significantly enriched in the methamphetamine group compared to controls. Key pathways impacted by methamphetamine included Herpes simplex virus 1 infection (*p* = 0.03), Human papillomavirus infection (*p* = 0.03), and Gastric cancer (*p* = 0.04). These pathways reflect the broad influence of methamphetamine on cellular processes, including cell proliferation and viral response mechanisms. Notably, the viral infection pathways involved DEGs such as *RT1-T24-3* and *Zik1*, highlighting methamphetamine’s potential role in modulating immune and viral response mechanisms within the brain. In addition to infection-related pathways, cell adhesion molecules (CAMs; *p* = 0.04) and the Hippo signaling pathway (*p* = 0.04) were enriched. Genes such as *Cdh1* and *Wnt6*, which regulate cell adhesion and growth, were upregulated, suggesting that methamphetamine exposure may alter cell adhesion dynamics and potentially contribute to neural plasticity alterations in the prefrontal cortex. Furthermore, the Ribosome pathway (*p* = 0.05) was significantly enriched, with genes like *Rpl35a* and *Rpl35al1* showing marked downregulation. This suggests methamphetamine-induced disruptions in ribosomal function and protein synthesis, potentially affecting neuronal integrity and function. Overall, the KEGG pathway enrichment analysis reveals that methamphetamine profoundly impacts pathways related to viral infection, cell adhesion, and protein synthesis. These findings are summarized visually in Figure 4, which provides a detailed representation of the significantly enriched pathways. 

### 2.5. Transcriptomic Changes in the Hippocampus: Differential Gene Expression

Our RNA-Seq analysis identified significant transcriptomic changes in the hippocampus following methamphetamine exposure, with 32 genes being differentially expressed between the methamphetamine and saline groups. These changes include 14 upregulated and 18 downregulated genes, with the majority being protein-coding genes (see Supplemental File Hip_SALVSHip_METH_Gene_differential_expression.csv for a complete list of DEGs with fold changes and *p*-values).

**Figure 5:**
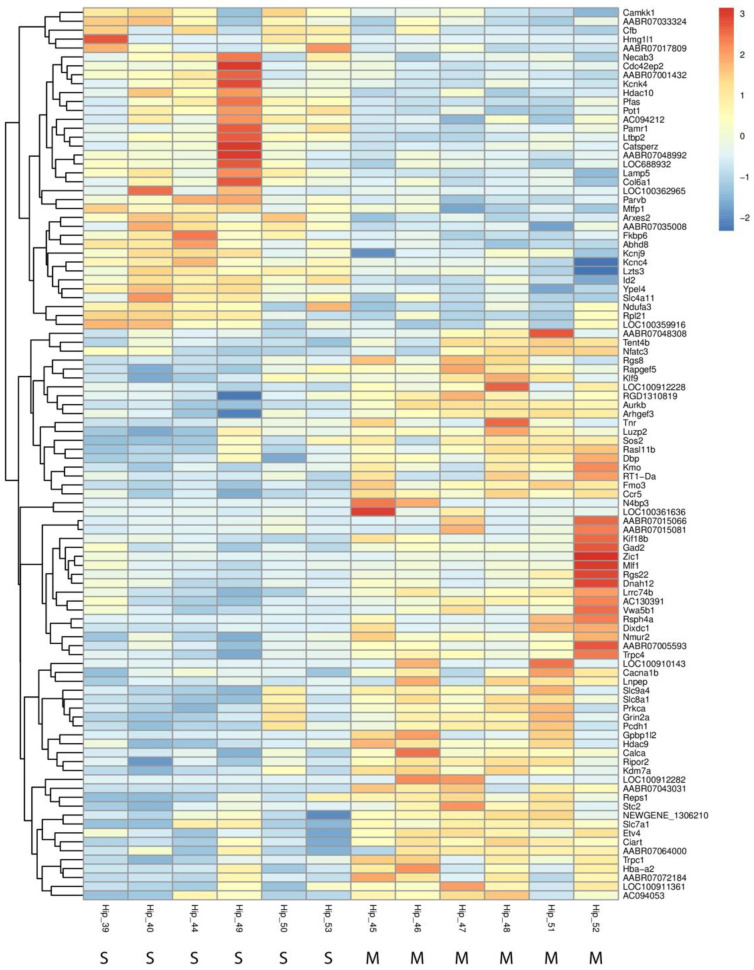
Heatmap of differentially expressed genes in the hippocampus following methamphetamine exposure. The heatmap showcases the most significantly upregulated and downregulated genes between methamphetamine-treated and control groups in the hippocampus. The heatmap illustrates individual sample variation, with color intensity representing the degree of gene expression change—red for upregulation and blue for downregulation. At the bottom of the figure, “M” indicates a sample from the METH group and “S” indicates a sample from the saline group.

Key upregulated genes include *Arxes2* (fold change = 2.05) and *Hmg1l1* (fold change = 6.09), both of which are implicated in chromatin remodeling and immune response activation. Additionally, the complement factor gene *Cfb* was upregulated, suggesting that methamphetamine may trigger immune-related processes within the hippocampus. Among the downregulated genes, *Rsph4a*, associated with ciliary function, and *Dixdc1*, involved in neuron differentiation and axon guidance, showed substantial reductions in expression. These changes likely affect neuronal signaling and hippocampal plasticity. Moreover, the downregulation of potassium channel genes *Kcnj9* and *Kcnk4* suggests potential alterations in neuronal excitability. Overall, the identified DEGs indicate that methamphetamine exposure affects a broad range of biological processes, including immune response, neurodevelopment, and neuronal signaling. These transcriptomic alterations are visually summarized in Figure 5, which highlights the most significantly changed genes across both up- and downregulated categories.

### 2.6. Gene Ontology Enrichment Analysis Reveals Differential Gene Expression in the Hippocampus Following Methamphetamine Self-Administration

**Figure 6:**
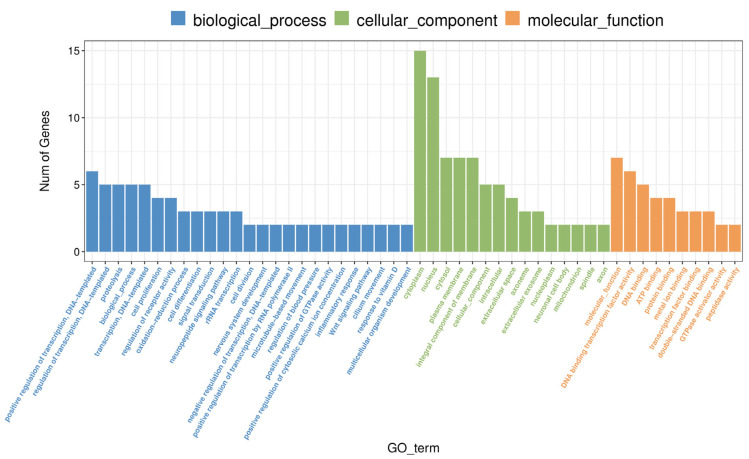
Gene ontology (GO) enrichment analysis of differentially expressed genes in the hippocampus following methamphetamine exposure. The chart illustrates significant GO terms enriched among differentially expressed genes, categorized into biological processes, cellular components, and molecular functions. Each bar represents a GO term, with the length corresponding to the number of genes.

To better understand the biological processes affected by methamphetamine exposure, we conducted Gene Ontology (GO) enrichment analysis on the differentially expressed genes (DEGs) identified in the hippocampus. Several GO terms were significantly enriched, revealing key insights into how methamphetamine impacts cellular function (see Supplemental File Hip_SALVSHip_METH_GO_enrichment_Gene.csv for a complete list of results). In the biological process category, significant downregulation was observed in genes related to “rRNA transcription” (GO:0009303) and “cell proliferation” (GO:0008283). Notable genes such as *AABR07015066* and *Aurkb* were involved in these processes, suggesting methamphetamine’s effect on reducing ribosomal RNA synthesis and inhibiting cell growth within the hippocampus. The molecular function category highlighted enriched terms like “DNA binding transcription factor activity” (GO:0003700) and “double-stranded DNA binding” (GO:0003690), with genes such as *Zic1* and *LOC100361636* significantly downregulated. These findings indicate that methamphetamine exposure alters transcriptional regulation and may suppress key pathways involved in DNA binding and transcription factor activity. Additionally, several enriched terms were related to calcium signaling and enzymatic activity, such as “calcium-dependent cysteine-type endopeptidase activity” (GO:0004198). The downregulation of genes like *Capn3* suggests methamphetamine-induced disruptions in calcium-dependent proteolytic processes, which may affect neuronal function and synaptic signaling. Overall, the GO enrichment analysis reveals methamphetamine’s broad effects on key biological processes in the hippocampus, including transcription regulation, cell proliferation, and calcium signaling. These findings are visually summarized in Figure 6, which illustrates the most significantly enriched biological processes.

### 2.7. KEGG Pathway Analysis in the Hippocampus: Differential Enrichment Under Methamphetamine and Saline Conditions

**Figure 7:**
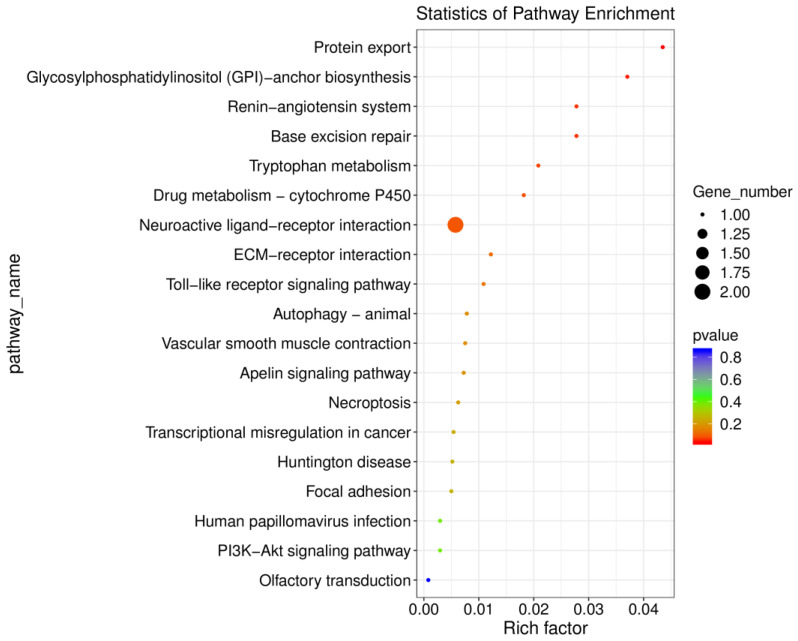
KEGG pathway enrichment analysis of differentially expressed genes in the hippocampus following methamphetamine exposure. This figure presents significantly enriched KEGG pathways in the hippocampus of methamphetamine-treated rats compared to controls. The point size represents the number of genes in each pathway, and the color reflects the *p*-value.

To further investigate the molecular pathways affected by methamphetamine in the hippocampus, we conducted KEGG pathway enrichment analysis. Several pathways were significantly enriched in the methamphetamine-treated group compared to controls (see Supplemental File Hip_SALVSHip_METH_KEGG_enrichment_Gene.csv for a complete output from these analyses). Key enriched pathways include “Protein export” (*p* = 0.03) and “Glycosylphosphatidylinositol (GPI)-anchor biosynthesis” (*p* = 0.04). These findings suggest that methamphetamine exposure enhances protein transport mechanisms and may disrupt cell surface signaling processes. Genes such as *Arxes2*, involved in protein export, and *LOC100910143*, associated with GPI-anchor biosynthesis, were central to these alterations. The “Base excision repair” pathway (*p* = 0.05) was also significantly enriched, with *Hmg1l1* showing substantial upregulation. This indicates that methamphetamine may induce DNA damage and activate repair mechanisms in the hippocampus. Additionally, the “Renin-angiotensin system” (*p* = 0.05) showed enrichment, highlighting possible impacts on blood pressure regulation through the downregulation of *Lnpep*. These enriched pathways reveal that methamphetamine affects critical biological processes related to protein transport, genomic stability, and cellular signaling. Figure 7 visually summarizes these pathway enrichments, illustrating the significant molecular changes in the hippocampus following methamphetamine exposure.

### 2.8. Secondary Analyses: Proof of Concept for Individual Gene Variability (PFC)

In this secondary proof-of-concept analysis, we assessed individual differences in gene expression within the prefrontal cortex (PFC) to evaluate the feasibility of detecting individual-level molecular signatures associated with METH exposure. To assess the effects of methamphetamine on specific genes in the prefrontal cortex, we performed a regression analysis using a negative binomial model. Several genes were found to be significantly upregulated in the methamphetamine group compared to controls. Notably, *Zic1* (adjusted *p* = 2.45 × 10^−6^), a transcription factor involved in brain development, and *AABR07015066* (adjusted *p* = 2.74 × 10^−6^), an uncharacterized gene, showed significant upregulation. These genes are associated with transcription regulation and neural development, suggesting that methamphetamine exposure may lead to widespread alterations in gene expression within the prefrontal cortex. Another gene, *AABR07015081* (adjusted *p* = 3.48 × 10^−4^), was also upregulated, further supporting the idea that methamphetamine influences transcriptional processes. These individual gene changes align with the broader findings from our pathway and GO analyses, which indicated that methamphetamine affects transcription regulation and cellular proliferation.

### 2.9. Secondary Analyses: Proof of Concept for Individual Gene Variability (Hippocampus)

In the hippocampus, our individual gene analysis revealed several key genes that were significantly upregulated in response to methamphetamine exposure. Among these, *Zic1* (adjusted *p* = 2.45 × 10^−6^), a transcription factor involved in brain development, and *AABR07015066* (adjusted *p* = 2.74 × 10^−6^), an uncharacterized gene, were particularly noteworthy. Both genes play important roles in transcription regulation and cellular signaling, suggesting that methamphetamine significantly impacts these critical processes in the hippocampus. Another upregulated gene, *AABR07015081* (adjusted *p* = 3.48 × 10^−4^), was implicated in similar regulatory functions, further reinforcing the idea that methamphetamine alters key pathways related to transcriptional activity and neurodevelopment. These findings align with our pathway and GO enrichment analyses, which demonstrated that methamphetamine influences transcription regulation and cellular proliferation in the hippocampus.

A key innovation of this study is the individual-level analysis of gene expression. Unlike most transcriptomic studies that aggregate data across groups, we show that assessing differential expression at the individual level is feasible. Despite the study’s small sample size, this method assesses individual molecular responses to METH use in rats, shedding light on biological variability in substance use behaviors and potentially inspiring more personalized substance use studies.

## 3. Discussion

This study provides novel insights into the molecular and behavioral effects of long-access METH self-administration, particularly focusing on neural substrates within the prefrontal cortex (PFC) and hippocampus. Using RNA sequencing, we identified a number of differentially expressed genes (DEGs) in both brain regions, many of which are involved in essential processes such as synaptic plasticity, neuroinflammation, and cellular metabolism. These changes show the profound impact of METH on neural function, as evidenced by the altered expression of genes regulating neurotransmission and mitochondrial function. In addition to the group-level changes, we observed individual variability in gene expression profiles that correlated with differences in behavioral demand for METH. Rats with higher economic demand for METH—a measure of motivation for drug-seeking—exhibited distinct gene expression patterns, particularly in pathways related to neural signaling and energy metabolism. These findings underscore the importance of integrating behavioral and molecular data to better understand the neurobiological mechanisms underlying substance use, both at the group and individual levels.

The group-level findings of this study align with previous research demonstrating that chronic methamphetamine (METH) exposure leads to significant transcriptional alterations in brain regions associated with cognitive control, decision-making, and reward processing. For example, prior studies have shown that METH exposure induces the expression of genes involved in synaptic plasticity and immune response in the prefrontal cortex (PFC) [5,31,32]. Similarly to our findings, the upregulation of genes associated with neuroinflammation and synaptic function has been observed, suggesting that METH disrupts normal neuronal connectivity and promotes a neurotoxic environment through inflammatory pathways. This neurotoxic effect is well-supported by studies showing that METH activates microglial cells, leading to the release of pro-inflammatory cytokines and chemokines, which exacerbate neuronal damage [33,34,35,36]. Additionally, increased expression of genes related to immune response and neuroinflammation, such as those involved in microglial activation and the complement cascade, has been observed following METH exposure in the striatum and PFC, further supporting our findings [36,37,38,39]. Although transcriptomic changes in the hippocampus have been less extensively studied in the context of METH exposure, our results suggest that the downregulation of potassium channel genes (*Kcnj9* and *Kcnk4*) may point to alterations in neuronal excitability and energy metabolism, which could represent a relatively underexplored consequence of chronic METH use.

The differential expression of genes involved in synaptic plasticity, neuroinflammation, and mitochondrial function observed in this study provides important insights into the neurobiological effects of chronic methamphetamine (METH) exposure. The upregulation of genes related to synaptic plasticity in the prefrontal cortex (PFC) suggests that METH may induce maladaptive changes in neuronal connectivity, potentially contributing to the compulsive drug-seeking behavior often seen in substance use. This is supported by previous studies indicating that METH exposure enhances synaptic activity, but in a way that disrupts normal cognitive functions such as decision-making and inhibitory control [1,40,41]. Notably, the upregulation of genes involved in transcriptional regulation and cell adhesion in the PFC, such as *Foxd1* and *Cdh1*, highlights how METH could be driving changes in gene networks that modulate synaptic structure and function, further promoting dysregulated signaling within the neural circuits related to substance use. In contrast, the downregulation of genes involved in mitochondrial and oxidative phosphorylation pathways, particularly in the hippocampus, suggests a potential mechanism for the cognitive deficits associated with long-term METH use. Mitochondrial dysfunction has been strongly implicated in neurodegenerative diseases and is increasingly recognized as a key factor in METH-induced neurotoxicity [19]. In line with these findings, our data suggest that the observed downregulation of genes such as *Pex5*, which plays a crucial role in peroxisomal protein import, and other metabolic genes may lead to impaired energy production and increased oxidative stress, contributing to neuronal damage. This is particularly relevant in the hippocampus, a brain region critical for learning and memory, which may explain the memory impairments often observed in chronic METH users [6,42]. The alterations in genes related to immune function, including the upregulation of *RT1-T24-3* in the PFC, are consistent with studies showing that METH induces neuroinflammatory responses, which exacerbate neuronal damage and synaptic dysfunction [34,38]. The combined impact of synaptic dysregulation, mitochondrial dysfunction, and inflammation suggests a complex, multidimensional mechanism by which METH exerts its long-term neurotoxic effects. While previous studies have established that METH disrupts these individual pathways, our findings advance this understanding by demonstrating that these molecular alterations are not only region-specific but also correlated with individual differences in drug-seeking behavior.

A key contribution of this study is the integration of behavioral economics with transcriptomic analysis to explore how individual differences in methamphetamine (METH) demand correlate with distinct gene expression profiles. This approach provides a deeper understanding of substance use vulnerability by demonstrating that individual behavioral variability—quantified through economic demand for METH—maps onto specific molecular pathways. Previous studies have highlighted the importance of considering individual differences in substance use, particularly in how genetic and neurobiological factors influence susceptibility to drug-seeking behaviors [43,44,45]. However, the majority of transcriptomic studies related to substance use have focused on group-level analyses, potentially masking the molecular signatures associated with individual variability in drug motivation. Our study advances the field by demonstrating that rats with higher METH demand, indicative of greater motivation to seek the drug, exhibited distinct upregulation of genes related to neural signaling and mitochondrial function. This finding suggests that the neurobiological mechanisms underlying substance use may differ among individuals, depending on their behavioral responses to drug exposure. The identification of specific genes, such as *Foxd1* and *Cdh1* in the prefrontal cortex, which were upregulated in rats with higher METH demand, suggests that variations in synaptic plasticity and cell adhesion processes could underlie individual differences in substance use vulnerability. This is consistent with previous research indicating that altered synaptic plasticity contributes to the compulsive drug-seeking behavior seen in substance use [11]. Additionally, the correlation between increased METH demand and mitochondrial dysfunction, as evidenced by the downregulation of genes involved in energy metabolism in the hippocampus, points to the role of cellular energetics in modulating substance use risk. Mitochondrial dysfunction has been implicated in neuropsychiatric disorders, including substance use, where energy deficits may influence an individual’s capacity to regulate stress and drug-seeking behavior [46]. By linking these individual behavioral and molecular profiles, our study highlights the value of personalized approaches in substance use research, aligning with the growing body of literature that advocates for individualized treatment strategies based on molecular and genetic differences [47]. This integration of behavioral economic models with gene expression data provides a novel framework for understanding heterogeneity in substance use, suggesting that therapeutic interventions could be tailored to target specific molecular pathways associated with higher vulnerability to METH use. While individual variability is increasingly recognized as a critical factor in substance use research, the molecular underpinnings of these differences remain poorly understood. Our findings contribute to closing this gap, emphasizing the need for future studies to expand upon these individual-level analyses to better inform personalized treatment approaches.

While this study provides novel insights into the molecular and behavioral mechanisms of methamphetamine (METH) substance use, several limitations must be acknowledged. First, the relatively small sample size, particularly in the individual-level analyses, may limit the generalizability of our findings. Although we observed significant correlations between METH demand and gene expression, future studies with larger cohorts are necessary to confirm these associations and improve statistical power. In addition, while our use of a long-access METH self-administration model better replicates the chronic drug exposure patterns observed in humans, it does not fully capture the complex environmental and psychological factors that contribute to substance use [43]. Future studies could expand upon this by incorporating stress models, social environments, or comorbid conditions, which are critical components of human substance use [48,49,50]. Another limitation is the focus on only two brain regions—the prefrontal cortex (PFC) and hippocampus—leaving out other regions critically involved in substance use, such as the nucleus accumbens and striatum. These areas are known to play key roles in the reinforcement and reward circuits affected by chronic METH use [51,52]. Future studies should examine gene expression changes in these additional regions to provide a more comprehensive understanding of METH’s effects on the brain. Furthermore, while our transcriptomic analysis identified differentially expressed genes associated with synaptic plasticity, mitochondrial function, and neuroinflammation, it is important to note that these findings are correlational in nature. Functional studies using gene knockdown, overexpression, or CRISPR-based approaches will be essential to establish causal relationships between specific genes and substance use-related behaviors. In addition, verification of our RNA-seq findings through independent methods, such as quantitative PCR (qPCR) or proteomic analyses, would strengthen the validity of the results and provide additional layers of molecular insight. Integrating multi-omics approaches, including proteomics and epigenomics, could offer a more holistic view of the molecular alterations induced by METH exposure and uncover additional therapeutic targets. Finally, our findings underscore the importance of personalized approaches to substance use research, where individual molecular profiles could guide the development of tailored therapeutic interventions. Future research should focus on identifying biomarkers of substance use vulnerability and resilience, potentially enabling the creation of personalized treatment strategies that account for genetic, epigenetic, and behavioral variability.

## 4. Materials and Methods

The experiment began with an initial phase of METH self-administration lasting 7 days, during which rats were trained to self-administer METH under a variable ratio schedule of reinforcement. This was followed by an economic demand assessment that measured how hard the rats were willing to work for METH across various fixed ratio reinforcement schedules, which were progressively increased between days (approximately 14 days). Subsequently, rats underwent an additional period of METH self-administration for at least seven more days to re-establish stable levels of METH self-administration. After completing these phases (approximately 28 days of METH exposure equated for all rats), brain tissue from the prefrontal cortex and hippocampus was collected and processed for RNA sequencing to identify differential gene expression.

### 4.1. Animals

Subjects were sixteen experimentally naïve adult (PD 60–90) male Sprague Dawley rats (250–300 g) purchased from Envigo (Indianapolis, IN, USA). Of the original sixteen, two rats did not survive the surgery, one lost catheter patency, and one succumbed to methamphetamine overdose, leaving twelve rats for the final experimental groups (six methamphetamine and six saline). Because of the novel nature and the experimental design of this study and the significant cost associated with RNA sequencing, only male rats were used in this study to minimize variance and to strengthen our ability to detect hypothesized effects. Rats were housed individually in a temperature- and humidity-controlled colony. Experiments were conducted during the light portion of a 12 h light/dark cycle (lights on at 7 a.m.). Following seven days of acclimation to the colony, rats were handled 2 min/day for five consecutive days. Water was freely available; access to chow was restricted after the acclimation period to maintain rats at 90% of their free-feeding body weight. This 90% target weight was increased by 2 g every four weeks from the beginning of the study. All procedures were in accordance with the Guide for the Care and Use of Laboratory Animals [53] and were reviewed and approved by the University of New Hampshire Institutional Animal Care and Use Committee (Protocol #160501).

### 4.2. Apparatus

Conditioning chambers (ENV-018MD; Med Associates, Inc.; St. Albans, VT, USA; 30.5 × 24.1 × 21.0 cm) were enclosed in a sound- and light-attenuating cubicle equipped with an exhaust fan. Each chamber had aluminum side walls and metal rod floors with polycarbonate front, back, and ceiling. Two nosepokes were mounted on each side of the chamber. A house light (two white 28 V, 100 mA lamps) was located 10 cm above the conditioning chamber ceiling. An infusion pump (PMH-100VS; Med Associates; St. Albans, VT, USA) fitted with a 5 mL syringe was located outside each enclosure. The infusion pump (PMH-100VS; Med Associates; St. Albans, VT, USA) for each chamber was located outside the sound-attenuating cubicle. A 5 mL syringe was connected to a swivel using Tygon^®^ tubing (AAQ04103; VWR; West Chester, PA, USA). Each swivel was coupled with a spring leash (C313C; Plastics One; Roanoke, VA, USA), which was suspended over the ceiling of the chamber on a balanced metal arm. Med Associates interface and software (Med-PC for Windows, version IV) were used to collect data and present programmed events.

### 4.3. Drugs

(+)-Methamphetamine hydrochloride was purchased from Sigma-Aldrich (St. Louis, MO, USA) and was dissolved in sterile 0.9% physiological saline. The methamphetamine dose was adopted from previous research [54,55].

### 4.4. Catheter Implantation Surgery

Rats were anesthetized using a 5% isoflurane and oxygen admixture for the induction phase and maintained at 1–3% thereafter. A polyurethane catheter (RJVR-23; Strategic Applications Inc.; Lake Villa, IL, USA) with a rounded tip and double suture beads, one secured internally and the other externally, was implanted into the right external jugular vein. The other end of the catheter was subcutaneously placed around the shoulder and exited below the scapula via a subcutaneously implanted polycarbonate back-mount access port (313-000BM; Plastics One Inc.; Roanoke, VA, USA). Immediately following the surgery, catheters were flushed with 0.2 mL of cefazolin (50 mg/mL) diluted in sterile heparinized saline (30 U/mL). Thereafter, these catheter flushes occurred daily until the end of the self-administration phase of the experiment to ensure patency. For pain management, rats were pretreated with meloxicam (2 mg/kg; SC) 5 min before the surgery. Meloxicam (2 mg/kg; SC) was also administered daily for three days after the surgery. Catheter patency was assessed when patency loss was suspected or upon completion of the self-administration phase of the study using an infusion of 0.05 mL xylazine (20 mg/mL; IV). This xylazine concentration produces clear motor ataxia within 5–10 s. Rats that did not exhibit noticeable motor ataxia within 5–10 s following xylazine infusion were considered non-patent.

### 4.5. METH Self-Administration and Assessment of Individual Demand for METH

Active nosepokes were assigned pseudo-randomly to each rat to ensure equal numbers of left and right active nosepokes among all rats. Rats spontaneously acquired METH self-administration. The beginning of each session was signaled by turning the house light off. Meeting a schedule requirement resulted in both nosepoke lights turning off and simultaneously turning the house light on for 20 s. During this 20 s signaled timeout, nosepoke entries were recorded but had no programmed consequences. All rats self-administered the exact dose of METH using a variation in infusion duration that was automatically calculated by the program based on their pre-session weight. Rats self-administered METH (0.05 mg/kg/inf; 10 h sessions during day phase) on a variable ratio (VR) 3 schedule of reinforcement (i.e., on average, every third response was followed by infusion; range = 1 to 5 presses) for seven consecutive days. Rats then were allowed to earn METH on the daily escalated FR schedules of reinforcement (1, 3, 5, 8, 12, 18, 26, 38, 58, 86, 130, 195, and 292). Each rat progressed through the range of FR schedules until failing to earn at least one infusion. Thereafter, rats were allowed to self-administer METH on the VR3 schedule of reinforcement for an additional 7 days or more, depending on the performance during the acquisition of economic demand phase, to equate the self-administration history for each rat (the number of self-administration sessions).

### 4.6. Tissue Collection, RNA Extraction, and Quality Control

The day after the final methamphetamine (METH) self-administration session, the prefrontal cortices—including the medial prefrontal cortex, prelimbic cortex, infralimbic cortex, and anterior cingulate cortex—along with the hippocampi and striata, were rapidly dissected on ice, immediately flash-frozen, and stored at −80 °C. This timing captures gene expression changes associated with active METH use rather than withdrawal effects, which would require additional timepoints to distinguish between transient and persistent transcriptional changes. The striatum samples were designated for a separate genomic study, whereas the prefrontal cortices and hippocampi were processed for this RNAseq study. Total RNA extraction was conducted using TRIsure reagent (Bioline, London, UK) following the manufacturer’s guidelines, consistent with methods detailed in our previous publications. The integrity of the RNA extracted from the nuclei was analyzed with the RNA IQ kit (Thermo Fisher Scientific, Waltham, MA, USA) on a Qubit 4.0 to determine the RNA Integrity Number (RIN). Approximately 0.85–3.15 µg of RNA per sample was sent on dry ice to LC Sciences (Houston, TX, USA) for RNA sequencing (see Table 1 for detailed sample information).

### 4.7. Library Construction and Sequencing

*RNA Sequencing Library Preparation:* The preparation of the poly(A) RNA sequencing library was conducted using Illumina’s TruSeq Stranded mRNA Sample Preparation protocol. Initially, RNA integrity was assessed using the Agilent Technologies 2100 Bioanalyzer. Subsequently, poly(A) tail-containing mRNAs were selectively purified employing oligo-(dT) magnetic beads, with the process being repeated for enhanced purity. Following purification, poly(A) RNA underwent fragmentation facilitated by divalent cation buffer at elevated temperatures. The construction of the DNA library was meticulously carried out, adhering to specified workflows. To ensure the quality and quantity of the sequencing library, analyses were performed using the Agilent Technologies 2100 Bioanalyzer High Sensitivity DNA Chip. Paired-end sequencing was executed on the Illumina NovaSeq 6000 system. One sample from the prefrontal cortex of a rat that self-administered methamphetamine was compromised during transport and was removed from further analyses.

### 4.8. Transcriptome Assembly and Bioinformatics Analysis

*Transcriptome Assembly:* Initial preprocessing involved the use of Cutadapt [56] and in-house perl scripts to cleanse the data of adapter sequences, low-quality bases, and undetermined bases. Sequence quality post-cleanup was verified through FastQC (version 0.10.1; http://www.bioinformatics.babraham.ac.uk/projects/fastqc/; accessed on 1 March 2020). The alignment of reads to the Rattus norvegicus genome (Ensembl release 96) was achieved using HISAT2 [57]. StringTie was then employed to assemble the mapped reads of each sample [58]. A comprehensive transcriptome was constructed by merging all sample-specific transcriptomes, utilizing perl scripts and gffcompare for comparison and consolidation. Expression levels of all transcripts were estimated using StringTie and edgeR [58,59].

*Differential Expression Analysis:* The analysis of mRNA expression levels was performed by StringTie, calculating FPKM values [58]. Differentially expressed mRNAs were identified based on a log2 fold change threshold (>1 or <−1) and statistical significance (*p*-value < 0.05) determined by the R package edgeR version 2016.09.29 [59].

### 4.9. Behavioral Data Analyses

The number of active and inactive levers during METH self-administration on the VR3 schedule of reinforcement was analyzed using mixed model ANOVA. Individual demand for METH was derived from the amount of methamphetamine consumed (mg/kg) over each FR schedule of reinforcement (Hursh and Silberberg, 2008). *Essential value* from the demand model was used to estimate individual demand for a reinforcer and was calculated from the nonlinear least squares regression model fit to the individual consumption data from each schedule of reinforcement using the following formula: $\log|Q|=|\log|Q_{0}|+|k(e^{-\alpha Q_{0}C}-1)$. In this model, *Q* represents reinforcer consumption, *Q*_0_ is consumption when the price is zero or free, *κ* is a constant for the range of demand, *e* is the base of the natural logarithm, *C* is the varying cost of each reinforcer, and *α* is the rate of decline in relative log consumption with increases in price. Maximum expenditure (*O_max_*) was calculated using the highest expenditure for each price or reinforcement schedule. The point of price where demand becomes elastic, and expenditure reaches a maximum (*O_max_*) is represented by *P_max_*. The *essential value* was calculated from the demand model using the following formula: $\mathrm{EV}=1/(100\;\times \;a\;\times \;k^{1.5})$. *EV* quantifies a reinforcer’s ability to maintain operant behavior amidst escalating behavioral costs and is often used to signify the intensity of demand or the value of a commodity. The economic demand curve analysis was performed using GraphPad Prism version 9, specifically employing a Prism template created by Hursh, S. R., and Roma, P. G. (2014) for exponential demand curve modeling, which was downloaded from https://ibrinc.org/software/ (accessed on 19 January 2022). This approach incorporates the use of an extra sum-of-squares F test, as recommended by the creators, to compare the fit of curves via the alpha (α) parameter, providing a preliminary evaluation of general effects. This template enables assessment at both grouped and individual levels. As recommended in the template, we employed the grouped approach for group-level analyses. For individual assessments, we generated individual economic demands. The Prism template offers an automatic selection feature for the shared kappa parameter. We utilized this feature in two instances: first, for grouped analyses, where kappa is shared among groups; and second, for individual economic demands, where kappa is shared among individuals. This automatic selection ensures optimal parameter fitting across different levels of analysis. Adopting this approach aligns our analysis with a rigorously validated framework, ensuring consistency with established research practices. Using this approach, we previously demonstrated that it is possible to plot individual demand curves for each subject and, most importantly, derive a single value of individual demand for a reinforcer that is based on performance over a range of schedules of reinforcement [27,30,60].

### 4.10. Proof of Concept: Individual Effects

As a secondary proof-of-concept analysis, we examined individual gene expression effects to explore the potential for capturing inter-individual variability in response to methamphetamine (METH) self-administration. The negative binomial model was used for individual gene assessments due to its suitability for analyzing count data, such as gene expression data (FPKM values). This model effectively handles zero-inflated data, which is common in gene expression data, particularly for lowly expressed genes, better than models like Poisson, which assume equal mean and variance. The negative binomial model provides interpretable coefficients, allowing interpretation of effect sizes and the direction of gene expression changes. Given the characteristics of gene expression data and the small sample size, the negative binomial model is a suitable choice for individual gene assessments. While the small sample size warrants cautious interpretation of the results, these findings can provide valuable insights for hypothesis generation and guide future investigations. However, additional studies with larger sample sizes will be necessary to validate and extend these results.

Thus, to assess the effects of methamphetamine self-administration on individual genes in the prefrontal cortex or hippocampus, we performed a regression analysis using the negative binomial model. The gene expression data (FPKM values) for each gene were modeled as a function of the *Essential Value* (*EV*), representing how hard each rat was willing to work for methamphetamine reinforcement. The analysis was conducted using the R programming language and the MASS library for fitting the negative binomial model [61]. For each gene, a separate regression model was fitted, and the coefficients, standard errors, z-values, and *p*-values were extracted. The *p*-values were then adjusted for multiple testing using the Benjamini–Hochberg correction method, which is a conservative approach to control the false discovery rate, ensuring that our findings are not inflated and remain reliable for further interpretation.

## Figures and Tables

**Table 1:** Sample information and behavioral economics parameters for individual subjects.

					Behavioral Economics Parameters			
Brain Area	Sample Name	Quality (RNA IQ)	Conc. (µg/µL)	Group Name	*EV*	*Q*_0_	*O_max_*	*P_max_*
Hip	Hip 45	7	2.3208	METH	0.82	19.00	22.77	3.69
Hip	Hip 46	7	2.4656	METH	0.79	2.80	22.06	24.28
Hip	Hip 47	8.2	1.48624	METH	2.64	6.00	73.54	37.77
Hip	Hip 48	7.6	1.95136	METH	1.81	7.20	50.43	21.58
Hip	Hip 51	7.4	1.80288	METH	1.01	8.90	28.24	9.78
Hip	Hip 52	9.1	0.85248	METH	4.22	5.10	117.66	71.10
PFC	PFC 45	7.6	2.14736	METH	0.82	19.00	22.77	3.69
PFC	PFC 46	7.2	2.6532	METH	0.79	2.80	22.06	24.28
PFC	PFC 47	7.4	2.3032	METH	2.64	6.00	73.54	37.77
PFC	PFC 48	8.3	1.44056	METH	1.81	7.20	50.43	21.58
PFC	PFC 51 *	7.6	2.29496	METH	1.01	8.90	28.24	9.78
PFC	PFC 52	7.6	3.15064	METH	4.22	5.10	117.66	71.10
Hip	Hip 39	6.8	2.19064	SAL	0.09	0.87	2.61	9.26
Hip	Hip 40	7.2	2.26464	SAL	0.00	0.74	0.11	0.46
Hip	Hip 44	7.2	2.26144	SAL	0.13	1.40	3.53	7.77
Hip	Hip 49	7.8	1.41472	SAL	0.15	2.30	4.15	5.56
Hip	Hip 50	7.8	1.70512	SAL	0.19	1.70	5.43	9.84
Hip	Hip 53	8.1	1.73056	SAL	0.07	0.52	1.91	11.31
PFC	PFC 39	8	1.51104	SAL	0.09	0.87	2.61	9.26
PFC	PFC 40	7.5	2.19352	SAL	0.00	0.74	0.11	0.46
PFC	PFC 44	7.5	2.18944	SAL	0.13	1.40	3.53	7.77
PFC	PFC 49	7.7	2.88296	SAL	0.15	2.30	4.15	5.56
PFC	PFC 50	7.9	1.84504	SAL	0.19	1.70	5.43	9.84
PFC	PFC 53	7.9	1.9628	SAL	0.07	0.52	1.91	11.31

Hip—hippocampus; PFC—prefrontal cortex; * sample excluded from the analyses because it was compromised during transport; METH—methamphetamine; SAL—saline. *EV*, *Q*_0_, *O_max_*, and *P_max_* are parameters derived from the exponential economic demand model of consumption.

**Table 2:** T-test comparisons of demand model parameters in rats self-administering methamphetamine vs. saline.

Parameter	*t*	df	*p*-Value	METH Mean	SAL Mean
*EV*	3.215	10	**0.0093**	1.879	0.105
*Q*_0_	2.956	10	**0.0144**	8.166	1.255
*P_max_*	2.064	10	0.0659	28.034	7.368
*O_max_*	3.215	10	**0.0093**	52.448	2.957

Significant comparisons are marked in bold in the *p*-value column.

## Data Availability

RNAseq data available in a supplement. Additional data is available upon request.

## Supplementary Materials

The following supporting information can be downloaded at: https://www.mdpi.com/article/10.3390/ijms26041400/s1.
